# Immune remodeling in chronic prostatitis: from microenvironment imbalance to targeted therapy

**DOI:** 10.3389/fmolb.2026.1876056

**Published:** 2026-09-08

**Authors:** Fulin Ma, Shiwei Song, Bin Zhang, Changfeng Yang, Chang Yu, Jinlong Yin, Linqi Liu, Dehui Chang

**Affiliations:** 1 Department of Urology, 940th Hospital of the Joint Logistics Support Force, Lanzhou, China; 2 College of Integrated Traditional Chinese and Western Medicine, Gansu University of Chinese Medicine, Lanzhou, China

**Keywords:** chronic pelvic pain syndrome, chronic prostatitis, immune homeostasis, inflammation, inflammatory microenvironment, targeted therapy

## Abstract

Chronic prostatitis/chronic pelvic pain syndrome (CP/CPPS) imposes a substantial clinical burden through persistent pelvic pain, lower urinary tract symptoms, sexual and reproductive dysfunction, recurrence, and variable responses to empirical therapy. Although infection, pelvic floor dysfunction and neurogenic mechanisms remain relevant, accumulating evidence indicates that persistent immune remodeling and inflammatory microenvironmental imbalance provide a unifying framework for disease chronicity. This narrative review synthesizes current evidence linking epithelial and stromal stress, ROS accumulation, cytokine and chemokine networks, NF-κB, JAK/STAT, NLRP3, HMGB1, HIF-1α and CXCR4 signaling with macrophage reprogramming, Th17/Treg imbalance, myeloid-derived suppressor cell activity and neuroimmune pain. Rather than treating these events as isolated pathways, we emphasize their crosstalk within a self-sustaining inflammatory circuit that connects local tissue injury with systemic metabolic and microbiota signals. We also discuss controversies surrounding IL-10 expression, immune-event causality and the limited direct evidence for immune-cell subset heterogeneity in CP/CPPS. Future work should integrate longitudinal cohorts, single-cell and spatial multi-omics, immune phenotyping and mechanism-oriented clinical trials to move CP/CPPS management from symptom control toward stratified immune intervention.

## 1 Introduction

Chronic prostatitis/chronic pelvic pain syndrome (CP/CPPS) is a common and disabling urological disorder characterized by chronic pelvic pain, lower urinary tract symptoms, sexual dysfunction, impaired semen quality, psychological distress and recurrent symptom flares (Schaeffer, 2008; Collins et al., 1998; Borgert et al., 2025; Magistro et al., 2016; Yani et al., 2022). Because many patients have no clearly identifiable infection and respond incompletely to empirical antibiotics, alpha-blockers, anti-inflammatory drugs or pelvic-floor intervention, CP/CPPS represents not only a pain syndrome but also a long-term clinical management challenge.

**FIGURE 1:**
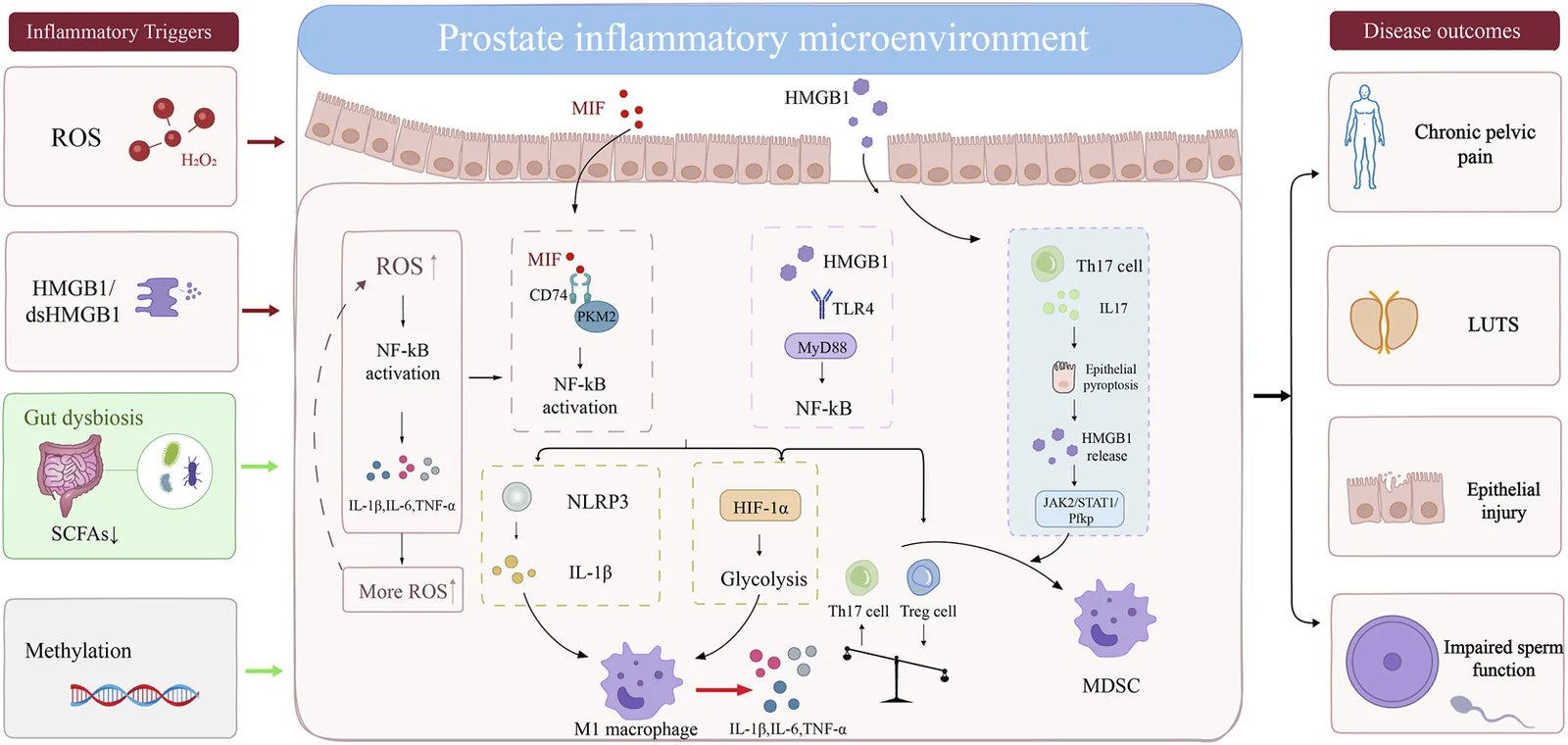
Immune microenvironment remodeling in CP/CPPS. Schematic diagram illustrating the bidirectional crosstalk between inflammatory triggers, epithelial/stromal stress, core signaling cascades, reprogrammed immune cell subsets, and final clinical disease outcomes in chronic prostatitis/chronic pelvic pain syndrome.

Research on CP/CPPS pathogenesis has evolved from an infection-centered model toward a broader framework that incorporates neuroimmune activation, pelvic-floor dysfunction, epithelial and stromal stress, systemic metabolism and microbiota-related signals. In this framework, the prostate is not a passive target of inflammation. Epithelial cells, stromal cells, infiltrating immune cells and soluble mediators form a dynamic microenvironment in which local damage, oxidative stress and immune activation reinforce one another (Tang et al., 2025; Ene et al., 2022). A key unresolved question is whether immune remodeling initiates CP/CPPS in susceptible patients or mainly maintains the chronic state after other triggers have acted. Current evidence supports both possibilities: macrophage activation, Th17/Treg imbalance, cytokine amplification and metabolic reprogramming have been observed in experimental models and clinical materials, yet their temporal order and causal hierarchy remain incompletely defined (Zhang et al., 2026; Dikov et al., 2026; Dalgleish and Liu, 2022).

As a narrative review, this article therefore organizes the field around an immune-remodeling axis: epithelial and stromal stress induces cytokine and chemokine release; these signals reprogram macrophages, T-cell subsets and immunosuppressive myeloid cells; the remodeled immune network establishes chronic inflammatory homeostasis; and this state contributes to pain, fibrosis, reproductive dysfunction and therapeutic resistance. By highlighting pathway-cell crosstalk, immune-cell heterogeneity, controversies and translational priorities, we aim to provide a mechanistic framework for future biomarker discovery and targeted immunomodulatory therapy.

## 2 The molecular basis of immune dysregulation

### 2.1 Epigenetic alterations

The immune dysregulation of CP/CPPS is not only reflected in the abnormal activation of inflammatory cells, but also stems from the disorder of upstream epigenetic regulation. Epigenetic studies on peripheral immune cells and prostate-related urine sediment in CP/CPPS patients further support the view that “immune regulatory deficiencies drive chronic inflammation”.

Thumbikat et al. analyzed the urine sediment of CP/CPPS patients and found that the local CD4^+^ T cycle-related transcripts of the patients were significantly elevated, accompanied by enhanced RORγT expression and decreased FoxP3-related regulatory features, suggesting that there is an adaptive immune imbalance in the prostate characterized by Th17 bias and insufficient Treg function. More importantly, targeted DNA methylation analysis revealed hypermethylation of the IL10 promoter in peripheral blood mononuclear cells (PBMC) and purified CD4^+^ T cells of CP/CPPS patients, and hypermethylation changes also occurred in the FOXP3 regulatory region of CD4^+^ T cells (Thumbikat et al., 2026). Research shows that CP/CPPS patients have extensive DNA methylation reprogramming, characterized by hypermethylation of the promoters of immune regulatory genes (such as IL10 and FOXP3), while hypomethylation of inflammation-related genes (such as TNFα, etc.), thus forming a molecular feature where pro-inflammatory enhancement and immunosuppressive weakening coexist. At the same time, The patient’s CD4^+^ T cells showed a significant Th1/Th17 bias, while the expression of the Treg-related transcription factor FOXP3 decreased, suggesting that the immune tolerance mechanism was impaired (Thumbikat et al., 2026).

In addition to DNA methylation, post-transcriptional regulation mediated by non-coding RNA may also be involved in the immune abnormalities of CP/CPPS. miR-155 is a key regulatory factor in the immune inflammatory response, significantly upregulated in the experimental autoimmune prostatitis (EAP) model, and accompanied by the activation of the TLR4/NF-κB pathway. The deletion of miR-155 can significantly alleviate pelvic pain sensitivity and prostatitis infiltration. Reduce pro-inflammatory factors such as IL-1β, TNF-α, IL-6, IFN-γ and IL-12, and simultaneously improve oxidative stress indicators such as SOD and GSH-Px (Fu et al., 2020).

### 2.2 Oxidative stress and reactive oxygen species (ROS)

ROS, as a natural product of aerobic metabolism in cells, participates in normal physiological processes such as cell proliferation, differentiation and signal transduction under physiological conditions. Under pathological conditions, the oxidative stress response triggered by excessive ROS production is a key link connecting chronic inflammation, tissue damage and abnormal immune cell function (Sharma et al., 2025). In the CP inflammatory microenvironment, the abnormal activation of immune cells can directly lead to the accumulation of pro-inflammatory factors and the imbalance of the antioxidant system, thereby inducing the massive generation of ROS and initiating the oxidative stress cascade response.

Excessive ROS causes multi-dimensional damage to prostate tissue: On the one hand, ROS can directly attack biological macromolecules such as DNA, proteins and lipids, causing DNA oxidative damage, protein denaturation and inactivation, and lipid peroxidation, destroying the structural integrity of prostate epithelial cells and stromal cells, and ultimately leading to an imbalance in the homeostasis of prostate tissue; On the other hand, ROS can act as a key signaling molecule to activate classic inflammation-related pathways such as nuclear factor-κB (NF-κB), further amplifying the inflammatory cascade response and forming a vicious cycle of “excessive ROS → inflammatory activation → further accumulation of ROS” (Figure 1) (Tang et al., 2025; Zhang et al., 2026). For example, the abnormal accumulation of ROS in prostate epithelial cells can specifically activate zinc finger protein 24 (ZNF24). Furthermore, it upregulates the expression of macrophage migration inhibitory factor (MIF), drives macrophages to polarize to M1 type, and ultimately aggravates the local inflammatory response (Zhang et al., 2026). Prdx5 promotes M1 polarization and apoptosis of prostate epithelial cells in a ROS-dependent manner through the TLR4/NF-κB axis, suggesting that the ROS-Prdx5-TLR4/NF-κB axis may be an important pathological chain connecting oxidative stress, macrophage polarization and apoptosis of prostate epithelial cells (Wu et al., 2025). In addition to the classic ROS/NF-κB inflammatory amplification mechanism, recent studies suggest that lipid metabolism may be an important upstream regulatory link for the persistence of oxidative stress injury. In the EAP model, the elevated expression of CXCR4 is not only associated with inflammatory cell infiltration, but also affects the desaturation process of polyunsaturated fatty acids (PUFA) by regulating the PPARγ/Fads2 axis. Inhibition or knockdown of CXCR4 can alleviate prostatitis in EAP mice and reduce lipid peroxidation products such as MDA and 4-HNE And reduce DNA damage and epithelial cell apoptosis; The inhibition of PPARγ can partially reverse the protective effect after CXCR4 silencing, suggesting that the metabolic changes of PUFA mediated by PPARγ/Fads2 are one of the important mechanisms by which CXCR4 regulates oxidative stress (Zhang et al., 2024).

It is worth noting that in prostate tissue, ROS is also widely regarded as the core molecule that links chronic inflammation with canceration. During the transformation process from prostatitis to prostate cancer, continuously elevated ROS can promote the malignant transformation of prostate tissue by activating oncogenic signaling pathways and inducing gene mutations, suggesting that ROS is not only a key pathological factor of CP, but also may be an important intermediate mediator for the progression of CP to prostate cancer (Tang et al., 2025).

### 2.3 Cytokines and signaling pathways

#### 2.3.1 Cytokines

The cytokine and chemokine network, as the core medium of intercellular communication in the inflammatory microenvironment, directly participates in the maintenance and amplification of chronic inflammation in CP by regulating the recruitment, activation and functional remodeling of immune cells. Among them, the synergistic effect of chemokines and pro-inflammatory factors is the key link in promoting the continuous activation of the inflammatory microenvironment of CP.

MIF can promote the assembly and activation of NLRP3 inflammatomes by activating the PI3K/AKT signaling pathway, thereby enhancing the maturation and secretion of pro-inflammatory cytokines such as IL-1β (Figure 1), and significantly intensifying the local inflammatory response of the prostate. This process suggests that MIF not only regulates the transcription of inflammatory genes through the traditional NF-κB pathway, but also participates in the amplification of the inflammatory cascade through the “PI3K/AKT-NLRP3 inflammasome” axis, constituting an important bridge connecting cytokine signaling and inflammasome activation. Therefore, the multi-pathway regulatory mechanism mediated by MIF plays a key role in maintaining the inflammatory microenvironment of CP and may become a potential therapeutic intervention target (Zhang F. et al., 2024).

The continuous high expression of key pro-inflammatory factors such as tumor necrosis factor-α (TNF-α), interleukin-1β (IL-1β), and interleukin-6 (IL-6) It is the core driving force for maintaining the chronic inflammatory state of CP (He et al., 2025; Murphy et al., 2015). In the experimental autoimmune prostatitis (EAP) rat model, the expression levels of IL-1β, IL-6 and TNF-α in the ventral prostate tissue were significantly increased. Moreover, its expression level is positively correlated with the degree of prostate tissue injury and the score of inflammatory symptoms (Chen et al., 2026). These pro-inflammatory factors can form a positive feedback loop of inflammatory amplification by activating classic inflammatory pathways such as NF-κB, further exacerbating prostate tissue damage. IL-6, as a core inflammatory mediator in the microenvironment of prostatic inflammation, can exert its functions through three signal transduction mechanisms: classical signaling (IL-6 binding to membrane-bound receptors), transsignaling (IL-6 binding to soluble receptors), and cluster signaling (signal transduction caused by receptor aggregation), thereby achieving dual functions of pro-inflammation and immune regulation (Ene et al., 2022).

It should be noted that interleukin-10 (IL-10), as an anti-inflammatory factor, its expression characteristics in CP remain controversial. Some studies have observed elevated IL-10 levels in chemotherapy-induced inflammation models, but this phenomenon may only be a compensatory response in specific situations. However, the expression pattern and regulatory role of IL-10 in CP models remain unclear and further research is needed to confirm it (He et al., 2025).

The expression pattern of interleukin-10 (IL-10) remains controversial in CP/CPPS, and this controversy probably reflects the biology of immune regulation rather than a simple technical inconsistency. IL-10 is usually regarded as an anti-inflammatory cytokine, but its measured level may increase as a compensatory brake after excessive TNF-α, IL-1β, IL-6 or IL-17 signaling, decline when Treg-related regulatory programs are epigenetically impaired, or remain locally insufficient despite peripheral elevation. This interpretation is supported by general IL-10 biology: IL-10 is produced by many innate and adaptive immune cells, acts through tightly regulated and sometimes compartmentalized signaling, and functions at different stages and anatomical locations of immune responses (Saraiva and O'Garra, 2010; Ng et al., 2013). In other words, IL-10 expression may represent three different states: an active resolution response, a failed regulatory response, or an immunosuppressive adaptation within a chronically inflamed tissue. Several mechanisms may underlie these apparently conflicting patterns. First, IL-10 is not restricted to one cell lineage; it can be produced by Treg cells, Th1/Th2/Th17 cells, CD8^+^ T cells, B cells, macrophages and dendritic cells (Saraiva and O'Garra, 2010; Maynard and Weaver, 2008). Therefore, changes in total IL-10 do not identify which cellular source is responsible or whether the cytokine is acting on antigen-presenting cells, effector T cells or regulatory populations. Second, IL-10 produced by FoxP3+ Treg cells, FoxP3- regulatory T cells and Tr1-like cells can function as a pivotal negative-feedback mechanism, but chronic antigen exposure can also induce IL-10-producing hyporesponsive regulatory cells, making IL-10 a marker of prolonged antigenic stimulation rather than simple inflammatory resolution (Ng et al., 2013; Saxena et al., 2015). Third, local prostate-derived specimens, urine sediment, semen and peripheral blood may capture different immune compartments; systemic elevation can therefore coexist with insufficient local immune resolution, a point consistent with the difficulty of harnessing systemic IL-10 therapy in autoimmune diseases despite its potent immunoregulatory activity (Wraith, 2003). Fourth, IL-10 also has context-dependent immunostimulatory effects, particularly on B cells and activated CD8^+^ T cells, which helps explain why high IL-10 is not always equivalent to disease suppression in autoimmune settings (Saxena et al., 2015; Groux and Cottrez, 2003). Finally, long-standing CP/CPPS may combine FOXP3/Treg dysfunction, IL10 promoter methylation or impaired IL-10 signaling with persistent pro-inflammatory stimulation (Thumbikat et al., 2026). Therefore, IL-10 should be interpreted together with cellular source, FOXP3/Treg stability, Th17 activity, pro-inflammatory cytokines, sample compartment and disease stage, rather than as a single-direction anti-inflammatory marker.

#### 2.3.2 Key signal pathways

In the CP inflammatory microenvironment, signaling pathways such as NF-κB, JAK/STAT, and TLRs/MyD88 do not operate in isolation but together form a core network that connects innate immune recognition, cytokine amplification, and phenotypic remodeling of immune cells. Among them, the NF-κB pathway holds a pivotal position. The imbalance of zinc homeostasis in the EAP model can activate the IKKβ/IκBα/NF-κB signaling, leading to a significant increase in pro-inflammatory factors such as IL-1β, IL-6 and TNF-α, and further aggravating tissue damage (Chen et al., 2026). Meanwhile, MIF induced by oxidative stress in epithelial cells can promote M1 polarization of macrophages through the CD74-PKM2-NF-κB axis, forming a positive feedback loop of “epithelial injury - macrophage activation - inflammatory amplification” (Zhang et al., 2026). These pieces of evidence suggest that the continuous activation of NF-κB is not only the molecular basis for maintaining the inflammatory microenvironment but also a key node connecting upstream stress signals with downstream immune effects.

In addition to NF-κB, JAK/STAT signaling participates in cytokine feedback and immune differentiation. STAT3 can synergize with hypoxia-inducible factor-1α (HIF-1α) to enhance glycolytic metabolism and sustain inflammatory programs, whereas inhibition of the STAT3/HIF-1α axis alleviates prostatitis in experimental settings (Wang C. et al., 2025). The TLR4/MyD88 axis senses microbial or damage-associated stimuli and promotes NF-κB p65 nuclear translocation (Zhihao et al., 2023). High mobility group box 1 (HMGB1), a damage-associated molecular pattern released by injured or pyroptotic epithelial cells, further engages the TLR4/NF-κB axis and amplifies macrophage infiltration and cytokine production (Kim et al., 2024). Thus, the key issue is not the presence of individual pathways, but their convergence into a shared network that converts tissue stress into immune-cell remodeling.

Metabolic reprogramming provides the functional link between pathway activation and immune-cell phenotype. HIF-1α acts as a hypoxia-metabolism-inflammatory polarization hub, whereas C-X-C chemokine receptor 4 (CXCR4) functions as a chemokine-axis node that can couple cell recruitment, oxidative stress and glycolysis. The Irf7/HIF-1α, CXCR4/c-MYC/PFKFB3 and dsHMGB1/Jak2/Stat1/Pfkp axes all indicate that enhanced glycolysis is not merely an energy adaptation but a driver of macrophage inflammatory polarization and stromal remodeling (Zhang Y. et al., 2024; Ma et al., 2025; Meng et al., 2025). These findings support a pathway-cell model in which ROS, NF-κB, JAK/STAT, HIF-1α, HMGB1 and CXCR4 jointly sustain chronic immune activation.

## 3 Immune cell reprogramming in chronic prostatitis

### 3.1 Macrophage polarization

Macrophages are among the most plastic immune cells in the CP/CPPS inflammatory microenvironment (Martinez and Gordon, 2014; Guo et al., 2020; Martinez and Motrich, 2025). Earlier studies often described this remodeling through the M1/M2 paradigm, but this binary framework is now insufficient. In CP/CPPS, macrophages should be viewed as a spectrum of inflammatory, tissue-remodeling, phagocytic, antigen-presenting and metabolism-associated states shaped by epithelial injury, cytokines, hypoxia, lipid metabolism and microbiota-derived signals. This spectrum-based interpretation is consistent with broader monocyte/macrophage literature showing multidimensional heterogeneity across classical, intermediate and nonclassical monocyte subsets, within-subset variation, disease-state effects and interindividual differences that may be transmitted to monocyte-derived macrophages (Williams et al., 2023; Appleby et al., 2013). It also aligns with single-cell immune-atlas evidence that ancestry, sex, age and population background alter immune-cell abundance and transcriptional traits, underscoring why CP/CPPS macrophage states should be resolved by cell-specific and compartment-matched approaches rather than inferred from M1/M2 markers alone (Kock et al., 2025).

Multiple inflammatory signals promote M1 polarization through synergistic effects, among which the NF-κB pathway constitutes the key regulatory axis. Epithelial cell-derived MIF directly induces the transformation of macrophages to the M1 phenotype through the CD74-PKM2-NF-κB axis (Figure 1) (Zhang et al., 2026); The CCL5/CCR5 axis maintains Stat1 phosphorylation and activates NF-κB signaling, promoting M1 macrophage polarization. The damage-related molecule, high mobility group protein B1 (HMGB1), can further amplify inflammatory signals through the TrAF6-dependent NF-κB pathway, forming a persistently activated state (Zhou et al., 2025). In addition, the CCL5/CCR5/SHP2 axis promotes macrophage polarization towards M1 type by maintaining Stat1 phosphorylation and activating NF-κB signaling, thereby exacerbating chronic prostatitis (Jin et al., 2024). These signals jointly construct a pro-inflammatory network centered on NF-κB, maintaining the continuous activation of macrophages. In addition to local inflammatory signals, metabolic regulation derived from the gut microbiota can also affect macrophage polarization. Eriocalyxin B can improve the integrity of the intestinal barrier, reshape the composition of the gut microbiota, and increase vitamin D3-related metabolic signals. *In vitro* experiments further confirmed that vitamin D3 can promote the transformation of macrophages from M1 phenotype to M2 phenotype (Yu et al., 2024).

Immune metabolic reprogramming is an important breakthrough direction in the research of prostatitis in recent years. Among them, HIF-1α is regarded as a key transcription factor that connects local hypoxia, inflammatory signals and changes in immune cell function. Under the background of persistent inflammation and immune cell infiltration, the prostate tissue may present a relatively hypoxic state, promoting the stabilization of HIF-1α and activating the downstream glycolytic program. HIF-1α not only upregulates the expression of various glycolycle-related enzymes, providing rapid energy support for pro-inflammatory immune cells, but also forms a synergistic network with signals such as STAT3 and NF-κB, further maintaining the transcription of inflammation-related genes. Interferon regulatory factor 7 (Irf7) enhances M1 polarization and exacerbates prostatitis and interstitial fibrosis by up-regulating the expression of hypoxia-inducible factor-1 α (HIF-1α) and promoting the transcription of glycolycle-related genes (Meng et al., 2025). Similarly, C-X-C receptor 4 (CXCR4) activates the downstream PI3K/AKT/mTOR pathway, upregulates c-MYC and its downstream key glycolytic enzyme PFKFB3, further enhances glycolytic levels to promote M1 polarization, and ultimately leads to prostatic fibrosis (Zhang Y. et al., 2024). More specifically, the disulfide bond form of HMGB1 released from pyroptosis prostate epithelial cells can upregulate the expression of phosphofructokinase platelet type (Pfkp), a key rate-limiting enzyme in glycolysis, through the Jak2/Stat1 signaling pathway, thereby enhancing the glycolytic metabolism of macrophages and promoting their polarization to the M1 phenotype (Ma et al., 2025). These pieces of evidence suggest that enhanced glycolysis is not only a metabolic feature of M1 polarization but also an important driving force for the persistence of inflammation.

This heterogeneity has practical implications. Inflammatory macrophages may amplify TNF-α, IL-1β and IL-6 signaling; tissue-remodeling macrophages may participate in fibrosis and extracellular-matrix deposition; metabolism-associated macrophages may be driven by HIF-1α-dependent glycolysis or lipid remodeling; and defective efferocytosis may impair inflammatory resolution. Evidence from related prostate inflammatory disorders and benign prostatic hyperplasia suggests lipid-rich and TREM2-or MARCO-associated macrophage states, but direct single-cell validation in CP/CPPS remains limited (Lanman et al., 2024). Future studies should therefore move beyond M1/M2 labeling and define macrophage subsets by transcriptomic state, spatial location, metabolic program and functional output.

Overall, macrophage remodeling in CP is manifested as a continuous process of “signal-driven - metabolic reprogramming - functional alteration”, with the core lying in the synergistic effect of the continuous activation of the NF-κB axis and the enhancement of glucose metabolism. This process not only maintains the inflammatory response but also exacerbates the disruption of the prostate microenvironment homeostasis by promoting epithelial cell damage and tissue fibrosis (Wu et al., 2025; Gao et al., 2019).

### 3.2 T cell imbalance

More and more research evidence indicates that CP/CPPS possesses the core characteristics of autoimmune diseases and is not merely an inflammatory lesion. The differentiation of T cell subsets has significantly shifted, showing an imbalance between Th17 cells and regulatory T cells (Treg), which has disrupted the dynamic balance between pro-inflammation and immunosuppression, directly promoting the progression of inflammation (Thumbikat et al., 2026). The balance among T cell subsets has a decisive influence on the inflammatory process (Awasthi and Kumar, 2019). Existing studies unanimously hold that the Th17/Treg imbalance is a hallmark of immune homeostasis disruption (Lv et al., 2025). In EAP models, Th17 cells are significantly elevated while Treg cells are decreased. This shift directly drives the persistence of the inflammatory response (Du et al., 2022; Liu X. et al., 2024). Further mechanism studies have shown that the Th17/Treg imbalance is not solely caused by the expansion of Th17 cells or the reduction of Treg cells, but may stem from the instability of Treg phenotypes induced by the inflammatory microenvironment. Liu et al. found in the EAP model that the NLRP3 inflammasome was significantly activated, accompanied by increased activation of caspase-1 and mature release of IL-1β. The level of IL-1β was positively correlated with the proportion of Th17 cells and Treg cells. The NLRP3 inhibitor MCC950 or IL-1R blockade can both reduce the ratio of Th17 and Treg, restore the relative level of Treg, and alleviate prostatitis (Liu X. et al., 2024). CP/CPPS belong to T-cell-mediated autoimmune diseases. Immune tolerance disorder and autoreactive lymphocyte activation jointly mediate persistent inflammatory responses and tissue damage (Chen et al., 2021). Clinical studies by Motrich et al. have demonstrated that Th17-mediated autoimmune responses play a core role in the persistence of inflammation and functional damage in CP/CPPS (Motrich et al., 2020). In addition, IL17 induced by T cells is crucial for the development of chronic pain in EAP (Ma et al., 2025). The CXCR4 signaling has also been proven to be an important upstream node connecting Th17 differentiation, oxidative stress and pain phenotypes. In EAP mice, the CXCR4 antagonist AMD3100 can significantly reduce prostatitis infiltration and pelvic mechanical pain sensitivity (Hu et al., 2026).

Dysbiosis of the intestinal flora is regarded as an important upstream factor driving abnormal T cell differentiation. The intestinal immune network activates pro-inflammatory mediators such as MCP-1, IL-1β, CXCL1, IGF-1 and their downstream pathways such as NF-κB, JAK/STAT, PI3K/Akt, ultimately promoting inflammation (Dovey et al., 2026). The reduction of short-chain fatty acids, especially propionic acid, can affect the differentiation direction of CD4^+^ T cells by regulating the activity of its receptor GPR43 and histone deacetylase (HDAC). In *in vitro* and *in vivo* experiments, supplementation of propionic acid can restore the Th17/Treg balance and significantly alleviate inflammation (Du et al., 2022). Besides short-chain fatty acids, bile acid metabolism has also been confirmed to be involved in the fine regulation of Th17 cell differentiation, and bile acids are involved in Th17 cell differentiation through the NLRP3-IL-17A signaling axis (Shao-Yu et al., 2025). The abnormal differentiation of Th17 cells is not only influenced by the overall structure of the intestinal microbiota, but also finely regulated by metabolic reprogramming and multiple signaling pathways. Firstly, at the level of metabolic regulation, Lycium barbarum polysaccharides (LBP) have been proven to inhibit the putrescine/TRAF/JAK/STAT signaling axis by regulating polyamine metabolism. Thereby inhibiting Th17 cell differentiation and alleviating the inflammatory response of experimental autoimmune prostatitis, suggesting the key role of metabolic-signaling pathway coupling in Th17 regulation (Wang X. et al., 2025). Meanwhile, external environmental factors can also regulate the Th17 response through the “gut microbiota-immune axis”. For instance, a high-salt diet can induce an imbalance in the intestinal flora and promote Th17 cell differentiation by activating the AHR/SGK1/FOXO1 signaling pathway, thereby exacerbating prostatitis. Similarly, alcohol intake can also drive metabolic pathways related to cholesterol biosynthesis by altering the intestinal microecological structure, further promoting Th17 cell differentiation and intensifying inflammatory responses (Du et al., 2024; Chen et al., 2025). These studies collectively indicate that the abnormality of Th17 cells is not a single immune event, but rather an important hub connecting environmental factors and immune imbalance through a multi-level regulatory network of the “gut microbiota-prostate axis”.

T-cell remodeling also extends beyond the Th17/Treg axis. Chronic inflammatory tissues may contain exhausted-like, tissue-resident memory-like or senescence-associated T-cell states with altered cytokine production, checkpoint expression and tissue retention. In prostate-related inflammatory and tumor microenvironments, CD8^+^ T-cell function can be constrained by checkpoint molecules such as PD-1 and LAG3 (Lanman et al., 2025; Shearer et al., 2026). However, EAP studies using CD8A-deficient mice suggest that CD8^+^ T cells may not be essential for disease induction or chronic pelvic pain development, whereas CD4^+^ Th1/Th17 responses appear more central to inflammation and pain (Salazar et al., 2024). These findings argue for a cautious interpretation: CD8^+^ T cells may modulate an established inflammatory environment rather than act as a universal initiating driver.

Accordingly, T-cell abnormalities in CP/CPPS should be analyzed as changes in both subset proportion and functional state. Th17 expansion, Treg instability, possible tissue-resident or exhausted-like populations and CD4+/CD8+ interactions with macrophages may jointly shape the persistence of inflammation. Because direct CP/CPPS single-cell and spatial evidence is still scarce, these proposed states should be treated as a working framework that requires validation by scRNA-seq, spatial transcriptomics, multiplex immunoimaging and longitudinal immune phenotyping.

### 3.3 Immunosuppression mediated by myeloid-derived suppressor cells

During persistent chronic inflammation, recruitment and expansion of immunosuppressive myeloid cells become an important feature of the inflammatory microenvironment. Myeloid-derived suppressor cells (MDSCs) are heterogeneous and are commonly divided into polymorphonuclear and monocytic subsets, which may suppress T-cell activation through arginine metabolism, ROS, nitric oxide, checkpoint signaling and cytokine networks. Direct evidence in CP/CPPS remains limited, but data from prostate inflammation, benign prostatic disease and prostate cancer suggest that MDSC-like programs may contribute to the paradoxical coexistence of persistent inflammation and local immunosuppression. The IKKβ/ARID1A/NF-κB axis can upregulate CXCR2 ligands and promote polymorphonuclear MDSC recruitment to prostate tissue (Li et al., 2022), providing a plausible bridge between inflammatory signaling and suppressive myeloid remodeling.

Functionally, MDSCs weakens the adaptive immune response by inhibiting the proliferation and effector functions of T cells, thereby creating a special state of “persistent inflammation and concurrent immunosuppression” locally. This dual regulatory model not only hinders the effective clearance of inflammation but also promotes the long-term existence of an imbalance between tissue damage and repair. In conclusion, the recruitment and functional exertion of MDSCs constitute an important bridge connecting inflammatory amplification and immunosuppression, and its role in the inflammatory microenvironment of CP cannot be ignored.

## 4 Inflammatory microenvironment remodeling

The inflammatory microenvironment in CP/CPPS is not a simple accumulation of isolated inflammatory mediators but a dynamic cellular network. A useful organizing sequence is: epithelial and stromal stress induces cytokine and chemokine release; these signals recruit and reprogram macrophages, T-cell subsets and suppressive myeloid cells; the remodeled immune cells produce ROS, HMGB1, MIF, IL-1β, IL-6, TNF-α and IL-17; and these mediators feed back onto epithelial, stromal and neural components to establish chronic inflammatory homeostasis. This circuit helps explain why CP/CPPS can persist after the initial trigger is no longer apparent.

### 4.1 Epithelial-stromal - immune cell interaction

In the CP inflammatory microenvironment, epithelial cells no longer merely serve as structural barriers but transform into active inflammatory regulatory nodes. Under inflammatory stimulation, prostate epithelial cells can regulate the behavior of surrounding stromal cells and immune cells by releasing cytokines, chemokines and exosomes (Zhao et al., 2021).

Zhao et al. found that the number of exosomes in the prostatic fluid of patients with type IIIA CP/CPPS increased significantly, and miR-155 was selectively enriched. These exosomes could be taken up in large quantities by prostatic stromal cells and induce the upregulation of IL-8 and TNF-α expression. However, after the destruction of the exosome membrane structure, its pro-inflammatory effect was significantly weakened, suggesting that miRNA delivery mediated by intact exosomes is a key link in inflammatory signal transduction (Zhao et al., 2021). In addition, the exosome miR-203a-3p derived from epithelial cells can target and inhibit the expression of DUSP5 in stromal cells, thereby activating the ERK1/2 signaling pathway and promoting the generation of monocyte chemoattractant protein-1 (MCP-1) (Calandra and Roger, 2003; Song et al., 2024). The increase of MCP-1 further promotes the recruitment of monocytes into prostate tissue and their differentiation into macrophages, thereby forming a tandem amplification mechanism of “epithelial cells - stromal cells - immune cells”. Meanwhile, stromal cells, as an important component of the inflammatory microenvironment, can secrete various chemokines and cytokines under the stimulation of inflammatory signals, maintaining immune cell infiltration and amplifying inflammatory responses. This interaction among multiple cell types transforms local inflammation from relying on a single cell source into a complex network driven by the coordinated efforts of multiple cell populations.

### 4.2 Positive feedback loop of inflammation

A core feature of the CP/CPPS inflammatory microenvironment is self-reinforcement. ROS activates NF-κB and promotes TNF-α, IL-1β and IL-6 expression; cytokines and chemokines recruit additional immune cells; infiltrating immune cells generate more ROS and damage-associated molecules; and Th17-derived IL-17 can induce epithelial pyroptosis and dsHMGB1 release, which further drives macrophage glycolysis and inflammatory polarization (Tang et al., 2025; Zhang et al., 2026; Ma et al., 2025). Thus, immune events should be interpreted as a feedback network rather than a linear sequence.

In addition, the damage-related molecules released during the inflammatory process (such as HMGB1) and MIF derived from epithelial cells can further promote the activation of macrophages and the release of pro-inflammatory factors, thereby enhancing the cascade amplification of inflammatory signals (Ma et al., 2025; Zhou et al., 2025). Meanwhile, IL-17 secreted by Th17 cells not only directly enhances the inflammatory response but also induces pyroptosis in epithelial cells and releases dsHMGB1, further driving macrophage metabolic reprogramming and inflammatory amplification (Figure 1) (Ma et al., 2025). The above processes jointly construct a “self-sustaining inflammatory circuit” driven by the interaction of ROS, cytokines and immune cells.

### 4.3 Immune regulation imbalance and the establishment of chronic inflammatory homeostasis

In a normal inflammatory response, there exists a dynamic balance between pro-inflammatory and anti-inflammatory mechanisms. However, in CP, this balance is disrupted, resulting in an immune state where pro-inflammation prevails and anti-inflammation is insufficient. Specifically, it is manifested as the continuous increase of pro-inflammatory factors such as TNF-α, IL-1β, and IL-6, while the anti-inflammatory regulatory mechanisms such as IL-10 and TGF-β are relatively insufficient, thereby resulting in the lack of effective termination signals for inflammation.

Adaptive immune imbalance plays a significant role in this process. The imbalance of the Th17/Treg ratio not only promotes the persistence of inflammation but also weakens the immune tolerance mechanism, keeping the inflammatory response at a low level for a long time but in a continuously activated state (Sharma et al., 2025; Awasthi and Kumar, 2019; Lv et al., 2025). Meanwhile, the accumulation of immunosuppressive cells such as MDSCs can inhibit the T cell effector function (Li et al., 2022), forming a special microenvironment of “persistent inflammation - coexistence of immunosuppression”. This dual state, on the one hand, hinders the clearance of inflammation, and on the other hand, prevents the immune system from being fully activated, thereby forming a stable but abnormal “chronic inflammatory homeostasis”.

The causal hierarchy among immune events is difficult to define because CP/CPPS is maintained by reciprocal loops rather than a single upstream-to-downstream pathway. Infection, autoimmunity, oxidative stress, pelvic-floor injury, microbiota dysbiosis and neurogenic inflammation may each initiate tissue stress in different patient subgroups. After the inflammatory network is established, however, ROS can activate NF-κB, NF-κB can increase cytokine and chemokine release, cytokines can recruit and polarize macrophages and T cells, Th17-derived IL-17 can promote epithelial injury and HMGB1 release, and macrophage-derived mediators can further damage epithelial and stromal compartments. These bidirectional interactions make it possible for the same event to be a consequence at one disease stage and a driver at another. Causality is further obscured by compartmental differences between local prostate tissue and peripheral samples, by the use of animal models that emphasize autoimmunity but do not fully capture chronic pain and pelvic-floor dysfunction, and by the fact that pain, fibrosis, urinary symptoms and immune-cell infiltration may not progress synchronously. Thus, the key mechanistic problem is not only identifying which immune events are altered, but determining when and in which patient subgroup each event becomes rate-limiting.

### 4.4 From local inflammation to systemic microenvironment remodeling

As the inflammation persists, the prostatic microenvironment gradually transforms from a local inflammatory state to a pathological system with changes in both structure and function. On the one hand, persistent inflammatory signals can induce phenotypic stabilization of immune cells, such as the long-term presence of pro-inflammatory macrophages and Th17 cells, enabling the inflammatory response to be maintained independently from the initial stimulus. On the other hand, cellular metabolic reprogramming (such as enhanced glycolysis) provides continuous energy support for these pro-inflammatory cells, thereby further consolidating the inflammatory state (Zhang Y. et al., 2024; Ma et al., 2025; Meng et al., 2025).

In addition, dysbiosis of the intestinal flora participates in the remodeling of the inflammatory microenvironment through the “gut-prostate axis”. Metabolites derived from the microbiota (such as short-chain fatty acids and bile acids) can affect T-cell differentiation and inflammatory signaling pathways, regulating the local immune status of the prostate at the systemic level (Du et al., 2022; Shao-Yu et al., 2025). This suggests that the CP inflammatory microenvironment does not exist in isolation but is the result of the interaction between local immune responses and systemic metabolism and microecological states. Microorganisms can affect the local immune status of the prostate through pathways such as short-chain fatty acids, bile acids, cholesterol metabolism, intestinal barrier integrity, LPS load, and Th17/Treg balance (Deng et al., 2025).

In summary, the remodeling of the inflammatory microenvironment in CP is a multi-level process driven by cellular interaction, signal amplification, immune imbalance and metabolic reprogramming, and the ultimate result is the formation of a self-sustaining chronic inflammatory system. This system not only explains the persistence and recurrence of the disease, but also provides an important theoretical basis for subsequent treatment strategies targeting immune homeostasis.

## 5 Clinical significance

### 5.1 Tissue remodeling and fibrosis

Chronic inflammatory stimulation can reshape the microenvironment of prostate tissue, with the core manifestations being fibrosis, stromal hyperplasia and somatic genomic mutations, etc (Gao et al., 2019). Inflammation-related cytokines, especially TGF-β and IL-6, play a key role in this process by activating fibroblasts and promoting extracellular matrix (ECM) deposition, driving the process of tissue fibrosis 7. Meanwhile, inflammatory mediators (such as IL-6) released by mast cells trigger prostatitis and directly stimulate the proliferation of prostate stromal cells (Han et al., 2023).

In the context of persistent inflammation, oxidative stress becomes an important hub connecting inflammation and tissue damage. The excessive production of ROS can not only induce DNA damage and gene mutations, but also activate multiple carcinogenic related signaling pathways (Tang et al., 2025). Meanwhile, the immunosuppressive microenvironment dominated by tumor-associated macrophages Tams and MDSCs can weaken the immune clearance ability of abnormal cells and promote epithelial-mesenchymal transition (EMT) by secreting multiple pro-transformation factors, thereby increasing the risk of tumor occurrence and progression (Zhang et al., 2025). It can be seen that the chronic inflammation in CP is not confined to the immune response itself, but drives tissue structure remodeling through a continuous immune-inflammatory network and, under certain conditions, constructs a continuous pathological spectrum of “inflammation - hyperplasia - tumor transformation”.

### 5.2 Reproductive dysfunction

The influence of CP-related inflammatory microenvironment is not only limited to the local prostate, but also causes systemic damage to male reproductive function through multi-dimensional regulatory networks. Current research has proposed a combined effect model of “inflammation - oxidative stress - endocrine imbalance - microbiota disorder”, revealing the full-cycle interference of the inflammatory microenvironment on spermatogenesis and function (Li et al., 2026).

At the cellular level, inflammatory cell infiltration and the release of local inflammatory factors can directly interfere with the process of spermatogenesis. Meanwhile, the continuous oxidative stress state leads to an imbalance in the antioxidant defense system. Excessive ROS can damage the sperm membrane structure and DNA integrity, thereby reducing sperm motility and survival rate. In addition, abnormal neuroendocrine regulation can disrupt the metabolic homeostasis of the reproductive system, and changes in the composition of the reproductive tract and intestinal flora further exacerbate sperm dysfunction.

Animal model studies provide direct evidence. In the EAP model, the inflammatory state significantly reduces sperm count, motility and mitochondrial membrane potential. Zinc supplementation intervention can alleviate the inflammatory response by inhibiting the IKKβ/IκBα/NF-κB signaling pathway and partially restore sperm functional parameters (Chen et al., 2026). CP/CPPS patients not only show decreased sperm motility, morphology and motility, but also significant sperm DNA damage and chromatin structure abnormalities at the molecular level (Berg et al., 2021). This result suggests that targeting inflammatory signaling pathways may become an important strategy for improving fertility disorders related to CP. Overall, CP-related immune imbalance disrupts the biogenic microenvironment through multi-pathway synergy, demonstrating an expansion from local inflammation to systemic reproductive function impairment.

### 5.3 Neuro-immune interactions and chronic pain

The typical clinical manifestations of CP, including chronic pelvic pain, frequent urination and urgent urination and other lower urinary tract symptoms (LUTS), are closely related to the continuous activation of local immune responses. Breser et al. proposed an integrated mechanism model, suggesting that the autoimmune response against prostate antigens is the core event of inflammatory initiation. This process is accompanied by the continuous recruitment of Th1 cells and various immune cells, thereby forming a chronic inflammatory microenvironment. On this basis, the release of inflammatory mediators and neuroactive molecules can induce peripheral and central nervous system sensitization, and then transform local inflammation into chronic pain signals (Breser et al., 2017). IL-1β, TNF-α and IL-6 can participate in the formation and maintenance of chronic pain by activating nerve endings and enhancing neural sensitization responses. Meanwhile, Th17-related cytokines (such as IL-17) can further amplify the inflammatory response and form a persistent inflammation-neural interaction network, suggesting the important role of the “neuro-immune axis” in chronic pain of CP. In the EAP model, mice showed significant pelvic tactile pain sensitivity. However, although TRPV1-deficient mice could still develop prostatitis infiltration, they did not exhibit the same degree of chronic pelvic pain. Local administration of TRPV1 antagonists could also alleviate the established pelvic pain sensitivity. This result suggests that TRPV1 is not a necessary condition for the occurrence of prostatitis, but a key node for the transformation of inflammatory signals into chronic pain and neurosensitization (Roman et al., 2020).

Inflammatory tissue edema, fibrosis and abnormal smooth muscle function can jointly affect the functions of the prostate and urethra, thereby leading to clinical manifestations such as frequent urination and urgent urination. In addition, immunosuppressive cells (such as MDSCs) and functionally remodeled macrophages can maintain a low but persistent inflammatory state, making the symptoms chronic and recurrent. It is worth noting that there is a certain correlation between immunophenotype and clinical manifestations. The increase in the levels of inflammatory factors such as IL-6, TNF-α and IL-17 is often positively correlated with the severity of the disease and symptom score, suggesting their potential application value in disease stratification and therapeutic effect evaluation. These findings further suggest that targeting neuroimmune interactions, especially the activation of immune cells and the sensitization pathways mediated by cytokines, may offer novel therapeutic opportunities that go beyond conventional anti-inflammatory methods. In recent years, intervention studies targeting the neuro-immune crossover node have further supported this view. Cannabidiol (CBD) not only reduces the expression of inflammation-related molecules such as IL-6, TNF-α, IL-8, COX-2 and NF-κB in the CP/CPPS model, but also inhibits TLR4/NF-κB signaling by activating the CB2 receptor. Thereby reducing the local inflammatory response of the prostate; More importantly, CBD exhibits a “post-activation desensitization” effect on TRPV1, enabling it to reduce pelvic pain sensitivity while suppressing inflammation (Piao et al., 2025).

## 6 Therapeutic strategies targeting immune homeostasis

The long-term persistence of CP is not caused by an abnormality of a single inflammatory factor, but rather a multi-level disruption of immune homeostasis composed of pro-inflammatory polarization of macrophages, Th17/Treg imbalance, amplification of oxidative stress, immune metabolic reprogramming, and abnormalities of the gut-prostate axis. Therefore, the treatment strategy should not merely focus on the control of downstream symptoms, but should carry out mechanism-oriented intervention around key immunopathological nodes. Based on the mechanisms mentioned earlier, the current potential therapeutic directions can be summarized into four categories: inhibiting macrophage-driven inflammatory amplification, restoring adaptive immune balance, regulating oxidative stress and immune metabolism, and reconstructing the homeostasis of the gut-prostate axis.

### 6.1 Targeting macrophage-driven inflammatory amplification

Macrophage M1 polarization is an important executive link for the sustainability of the CP inflammatory microenvironment. Therefore, inhibiting the maintenance of pro-inflammatory macrophages is one of the key strategies to block the positive feedback loop of inflammation. Existing studies suggest that MIF derived from epithelial cells can drive macrophages to transform into the M1 phenotype through the CD74-PKM2-NF-κB axis and promote the release of pro-inflammatory factors such as TNF-α, IL-1β and IL-6 (Zhang et al., 2026). Therefore, MIF inhibitors (such as ISO-1) or CD74 blocking antibodies may weaken M1 polarization and its inflammatory amplification effect by interfering with epithelial-macrophage paracrine communication.

Apart from the MIF-CD74 axis, NF-κB remains a convergent hub for macrophage-related inflammatory responses. Upstream inputs including ROS, HMGB1, CCL5/CCR5/SHP2 and TLR4/MyD88 can all maintain pro-inflammatory macrophage states (Chen et al., 2026; Wang C. et al., 2025; Martinez and Gordon, 2014; Li et al., 2026). Because NF-κB is also required for host defense, tissue repair and cell survival, future strategies should prioritize selective modulation of upstream axes or cell-specific delivery rather than broad long-term inhibition.

In addition, macrophage-associated kinases may also become new intervention nodes. For instance, PIM kinase inhibition can reduce inflammasome activity and improve the immune microenvironment, and has shown certain potential in the immune microenvironment of prostate tumors (Huang et al., 2026; Clements et al., 2025). Although this evidence mainly comes from tumor-related studies, it suggests that the “macrophage kinase - inflammasome - immunosuppression” axis in a chronic inflammatory state may have therapeutic value across disease scenarios. In the future, its safety, efficacy and applicable patient subgroups still need to be further verified in the CP/CPPS model.

### 6.2 Restore adaptive immune balance

Adaptive immune imbalance, especially the abnormal Th17/Treg ratio, is an important sign of the disruption of CP immune homeostasis. Excessive activation of Th17 cells can amplify local inflammatory responses through inflammatory mediators such as IL-17, while the decline of Treg function weakens anti-inflammatory regulatory mechanisms such as IL-10 and TGF-β, resulting in a lack of effective negative feedback (Jin et al., 2024; Lanman et al., 2024) in inflammation. Therefore, inhibiting the abnormal differentiation of Th17 or restoring the immune regulatory function of Treg is an important direction for rebuilding adaptive immune balance.

Mesenchymal stromal cell (MSCs) therapy offers a potential strategy for restoring immune homeostasis. Previous studies have shown that MSCs can alleviate CP/ CPPS-related inflammation by regulating systemic immune responses, increasing the proportion of Treg, and promoting the secretion of anti-inflammatory factors (Liu H. et al., 2021). It is worth noting that IL-1β pretreatment can enhance the immunomodulatory ability and targeted migration ability of MSCs, and further improve the therapeutic effect by down-regulating the NF-κB and JNK/MAPK pathways (Liu H. et al., 2021). This suggests that cell therapy is not merely about supplementing exogenous immunomodulatory cells, but can be functionally optimized through inflammatory pretreatment, engineering modification or targeted delivery.

In addition to cell therapy, directly regulating the pathways related to Th17 differentiation also has potential value. For instance, NLRP3-mediated IL-1β can be involved in Th17/Treg imbalance (Liu X. et al., 2024), while the polyamine metabolism-related TRAF/JAK/STAT axis has also been confirmed to be involved in Th17 differentiation regulation (Wang X. et al., 2025). Therefore, future therapeutic strategies can be further developed around the axes of “inflammasome - IL-1β -TH17”and “metabolite - JAK/STAT - Th17”. However, at present, most of the relevant evidence is concentrated on animal models. Before clinical application, issues such as dosage, safety, long-term immunosuppression risk and patient stratification still need to be addressed.

### 6.3 Regulate oxidative stress and immune metabolism

Oxidative stress is a core hub connecting tissue damage, inflammatory amplification and immune cell remodeling. Excessive accumulation of ROS in the CP inflammatory microenvironment can directly damage DNA, proteins and lipids, and induce the release of pro-inflammatory factors (Tang et al., 2025; Zhang et al., 2026) by activating signaling pathways such as NF-κB. Therefore, antioxidant therapy may not only alleviate tissue damage but also indirectly block the positive feedback loop of “ROS - NF-κB - cytokines - immune cell infiltration”.

The natural product Dioscin can inhibit the expression of TLR4 and the phosphorylation of NF-κB p65, reduce the accumulation of ROS and MDA, restore the activities of SOD and CAT, and improve the pathological damageof prostate tissue (Long et al., 2024). Oxidative stress intervention is not only limited to directly eliminating ROS, but also can reduce lipid peroxidation susceptibility by regulating the composition of lipid substrates. The metabolic alterations of PUFA mediated by the CXCR4-PPARγ-Fads2 axis suggest that targeting the fatty acid desaturation process or supplementing specific ω-3 PUFA may become metabolic immune intervention strategies in addition to antioxidant therapy (Zhang et al., 2024). Zinc supplementation intervention provides experimental evidence for regulating oxidative stress and inflammatory signals. In the EAP model, zinc can reduce the levels of IL-1β, IL-6 and TNF-α by inhibiting the IKKβ/IκBα/NF-κB signaling pathway, and improve inflammation-related tissue damage and sperm function parameters (Chen et al., 2026). This indicates that the regulation of trace elements may achieve dual benefits by restoring REDOX homeostasis and inhibiting inflammatory signals. However, whether zinc intervention is applicable to different clinical subtypes of CP/CPPS, as well as its optimal dosage, treatment course and long-term safety, still require further clinical verification.

Immune metabolic reprogramming is another therapeutic entry point. Instead of repeatedly targeting individual mediators, future strategies could focus on the HIF-1α-centered glycolytic program and its upstream regulators, including Irf7, CXCR4 and HMGB1-related signaling (Zhang Y. et al., 2024; Ma et al., 2025; Meng et al., 2025). Because glycolysis is essential for normal immune and stromal functions, translation will require local delivery, patient stratification, combination therapy or transient intervention windows.

### 6.4 Target the gut-prostate axis

Dysbiosis of the gut microbiota participates in the persistence of CP inflammation by influencing immune metabolism and T cell differentiation, and has gradually attracted attention as a systemic intervention target in recent years. Studies have shown that short-chain fatty acids, especially propionic acid, can regulate the differentiation of CD4^+^ T cells through GPR43 and histone deacetylase. Supplementing propionic acid can restore the Th17/Treg balance and alleviate EAP inflammation (Du et al., 2022). This suggests that supplementing short-chain fatty acids, regulating the structure of the microbiota or restoring the metabolic function of the microbiota may help to rebuild adaptive immune homeostasis. In addition to short-chain fatty acids, bile acid metabolism, cholesterol biosynthesis, and alterations in the microbiota induced by high salt or alcohol can also affect Th17 differentiation through the NLRP3–IL-17A, AHR/SGK1/FOXO1 and related metabolic pathways. (Shao-Yu et al., 2025; Du et al., 2024; Chen et al., 2025). The research on astaxanthin (AST) further provides experimental evidence for microbiota targeted intervention. AST enhances intestinal barrier function by upregulating the abundance of Ackermansia mucinophilus, thereby reducing the infiltration of CD4^+^T cells and macrophages in the prostate stroma and inhibiting the expression of inflammatory mediators, thus alleviating the tactile sensitivity response in the pelvic region (Liu Y-F. et al., 2024). Berberine can partially restore the homeostasis of the microbiota and may alleviate prostatitis in mice by regulating the anti-inflammatory and antioxidant properties of key microbiota (Tian et al., 2024). This indicates that the gut-prostate axis is not a single change in the microbiota, but a complex regulatory network composed of microbiota composition, metabolite profiles and immune signals. Future microecological intervention should not be limited to the broad supplementation of probiotics, but should be precisely designed in combination with the patient’s microbiota characteristics, metabolite deficiencies and immunophenotypes. CP/ CPPS-related microecological abnormalities are not limited to the intestinal tract. They may also involve the urinary tract, semen and oral microbiota. The local microecology of the prostate itself may also directly participate in the regulation of immune homeostasis. Healthy prostate-derived *Streptococcus* epidermidis strains reduce IL-17 and CD4^+^ T cell responses through PDL1/PDL2 and related mechanisms, suggesting that prostatic symbiotic bacteria are not merely a colonization background but may act as active regulators of local immune tolerance and pain control (Murphy et al., 2017). Therefore, future microecological intervention strategies should not only focus on the reconstruction of intestinal flora, but also take into account the local symbiotic bacteria of the prostaterogenital tract and their immune active molecules.

Furthermore, multi-omics studies suggest that alterations in the gut microbiome, transcriptome, and methylation may jointly be involved in the occurrence and development of chronic non-bacterial prostatitis (Prakash et al., 2024). Metabolites derived from the microbiota may also affect the progression of prostate diseasesthrough signals such as PI3K/Akt and AGE-RAGE (Liu J. et al., 2021; Xu et al., 2026). Therefore, the gut-prostate axis is expected to become an important therapeutic entry point connecting local inflammation with systemic immune metabolic abnormalities. However, current related research still mainly focuses on animal experiments and association analyses. In the future, prospective clinical studies are needed to verify the actual improvement effects of microecological intervention on pain, urination symptoms, inflammatory indicators, and recurrence rates.

### 6.5 From symptom control to precise immune intervention

At present, the clinical treatment of CP/CPPS still mainly relies on non-steroidal anti-inflammatory drugs, alpha receptor antagonists, empirical use of antibiotics, pelvic floor treatment and local intervention, etc. These strategies have certain value in alleviating pain, urination symptoms or local discomfort, but most of them act on downstream manifestations of the disease and are difficult to systematically correct the imbalance of immune homeostasis. Therefore, the future treatment concept needs to gradually shift from “symptom relief” to “precise intervention guided by immunophenotypic stratification”.

Specifically, a stratified treatment framework can be established based on the dominant immune characteristics of the patients. For example, for patients mainly characterized by M1 macrophage activation and enhanced NF-κB signaling, targeting MIF-CD74, TLR4/MyD88 or NF-κB-related pathways can be given priority. For patients with a significant imbalance between Th17 and Treg, it is possible to explore restoring Treg function, inhibiting Th17 differentiation or regulating the metabolites of the intestinal flora. For patients with prominent oxidative stress and reproductive function impairment, antioxidant treatment, zinc homeostasis regulation and immunometabolic intervention can be regarded as key directions. For patients with obvious immunosuppressive characteristics or accompanied by the risk of tissue remodeling, attention should be paid to the MDSCs-like network, macrophage functional status and immune checkpoint related pathways.

Overall, the future treatment of CP is likely to not rely on a single target but rather require a multi-module combined intervention, that is, a comprehensive strategy of “anti-inflammation + immune regulation + antioxidation + microecological reconstruction”. Meanwhile, the Th17/Treg ratio, macrophage polarization markers, inflammatory factor profiles such as IL-6/TNF-α/IL-17, ROS levels, and characteristics of microbiota metabolites may all become candidate biomarkers for patient stratification and therapeutic efficacy prediction. Only by integrating mechanism research, immune typing and clinical validation can the treatment of CP/CPPS truly move from empirical management to precise immune regulation.

## 7 Discussion

This review supports a central interpretation: in many patients with CP/CPPS, persistence of symptoms is likely maintained by an immune-remodeling network rather than by a single pathogen, cytokine or immune-cell type. The most plausible sustaining core consists of macrophage inflammatory polarization, Th17/Treg imbalance, ROS-mediated epithelial and stromal stress, NF-κB/JAK-STAT/NLRP3 pathway convergence, HIF-1α-dependent glycolysis and microbiota-related immune modulation. These axes are clinically important because they connect tissue inflammation with pain, fibrosis, reproductive dysfunction and variable responses to conventional therapy.

The main conceptual challenge is causality. Current studies often demonstrate association, pathway activation or therapeutic reversal in animal models, but they rarely define the temporal order of immune events in patients. The underlying reason is that several immune modules can activate one another: epithelial injury and ROS activate NF-κB; NF-κB-driven cytokines recruit and polarize macrophages; macrophage-derived mediators and Th17 cytokines aggravate epithelial damage; HMGB1 and CXCR4 amplify cell recruitment and metabolic remodeling; and IL-10-related regulatory responses may either restrain this process or mark failed resolution. Macrophage activation, Th17/Treg imbalance, IL-10 changes, CXCR4 activation, HMGB1 release and HIF-1α-dependent glycolysis may therefore be initiating drivers in one context but secondary adaptations in another. This explains why cross-sectional studies can repeatedly identify the same immune abnormalities without proving which event is primary. Longitudinal sampling, cell-specific perturbation, compartment-matched specimens and immune-phenotype stratification are therefore required before these molecules can be treated as universal causal targets. This is especially important for IL-10, because the same cytokine can mediate negative feedback, support regulatory T-cell function, reflect chronic antigen exposure or exert immunostimulatory effects depending on cellular and tissue context (Ng et al., 2013; Saxena et al., 2015; Wraith, 2003; Groux and Cottrez, 2003).

Another limitation is evidence imbalance. Th17/Treg dysregulation, pro-inflammatory cytokines, macrophage activation and NF-κB-related amplification are supported by both experimental models and clinical observations. By contrast, MDSC-mediated immunosuppression, exhausted-like T-cell states, macrophage subset heterogeneity, immune checkpoint involvement and gut-prostate axis mechanisms are still supported mainly by animal models, omics associations or evidence from adjacent prostate diseases (Hagiwara et al., 2022). These hypotheses are valuable, but the manuscript now frames them as testable mechanisms rather than established clinical facts.

Future research should become more stratified and technically precise. First, CP/CPPS cohorts should be divided by immune phenotype instead of being analyzed as a single disease entity, using candidate markers such as Th17/Treg ratio, macrophage-state markers, IL-6, TNF-α, IL-1β, IL-17, IL-10, ROS, HIF-1α-related metabolic indicators and microbiota-derived metabolites. Second, longitudinal clinical cohorts should test whether these markers precede symptom flares, tissue remodeling or therapeutic response. Third, single-cell transcriptomics, spatial transcriptomics, multiplex immunoimaging, metabolomics and microbiomics should be integrated to map epithelial-stromal-immune-neural crosstalk in local and surrogate samples. Fourth, clinical trials should move beyond single symptom endpoints and combine NIH-CPSI scores, pain sensitivity, urinary symptoms, semen parameters, inflammatory profiles, immune phenotypes, microbiota features and recurrence rates.

Translationally, immune-targeted therapy for CP/CPPS is unlikely to succeed through one broad anti-inflammatory agent. A more realistic strategy is modular intervention: blocking macrophage inflammatory amplification in macrophage-dominant disease, restoring adaptive immune balance in Th17/Treg-dominant disease, targeting oxidative stress and immunometabolism in metabolically inflamed disease, and reconstructing microbiota-derived immune regulation in gut-prostate-axis-dominant disease. Such approaches will require biomarker-guided patient selection and careful avoidance of non-selective immunosuppression.

In summary, CP/CPPS should be considered a heterogeneous syndrome in which immune remodeling can maintain a chronic inflammatory microenvironment and link local prostate injury with systemic metabolic and neuroimmune signals. Clarifying immune-cell heterogeneity, resolving causal relationships and validating stratified therapeutic targets are the key steps needed to move the field from empirical symptom management toward mechanism-guided precision intervention.

## Data Availability

The original contributions presented in the study are included in the article/Supplementary Material, further inquiries can be directed to the corresponding author.
